# Photon-counting CT vs V/Q SPECT for lobar perfusion quantification in chronic thromboembolic pulmonary hypertension

**DOI:** 10.1007/s00330-026-12547-y

**Published:** 2026-04-24

**Authors:** Matthias M. V. Moeskes, Thorsten Derlin, Anna M. Hunkemöller, Cornelia Schäfer-Prokop, Norman Kornemann, Jan W. Eckstein, Bernhard Meyer, Jens Vogel-Claussen, Frank K. Wacker, Hoen-oh Shin

**Affiliations:** 1 Institute of Diagnostic and Interventional Radiology, Hannover Medical School, Hannover, Germany; 2 Nuclear Medicine Clinic, Hannover Medical School, Hannover, Germany; 3 Institute for Radiology, Nuclear Medicine, and Molecular Imaging, Heart and Diabetes Centre North Rhine-Westphalia, Ruhr University Bochum and Medical Faculty OWL (University of Bielefeld), Bad Oeynhausen, Germany; 4 Department of Radiology, Radboud University, Nijmegen, The Netherlands; 5 Department of Radiology, Meander Medical Center, Amersfoort, The Netherlands; 6 Biomedical Research in Endstage and Obstructive Lung Disease Hannover (BREATH), German Center for Lung Research (DZL), Hannover, Germany

**Keywords:** CTEPH, Lung perfusion, Photon-counting CT, Quantitative perfusion analysis, V/Q-SPECT

## Abstract

**Background:**

Ventilation/perfusion single-photon emission computed tomography (V/Q-SPECT) is the standard first-line imaging method for chronic thromboembolic pulmonary hypertension (CTEPH), but it lacks anatomical detail. Photon-counting computed tomography (PCCT) enables high-resolution assessment of perfusion, vasculature, and parenchyma in one scan, potentially improving diagnostic accuracy.

**Objectives:**

To compare quantitative lobar lung perfusion between PCCT and V/Q-SPECT in patients with suspected or confirmed CTEPH.

**Materials and methods:**

This retrospective single-centre study included twenty-three patients (ten females, thirteen males; mean age 67.9 ± 10.7 years). The median interval between PCCT and V/Q-SPECT imaging was 3 days (range: 0–11 days). Lung perfusion was analysed on a lobar basis using PCCT-derived perfused blood volume (PBV) maps and V/Q-SPECT perfusion images. Data were normalised using a *z*-score approach based on the 95% confidence interval. Lobar segmentation was performed with TotalSegmentator. Pearson correlation and Bland–Altman analyses compared lobar and whole-lung perfusion metrics. Perfusion defect volumes were quantified from normalised maps.

**Results:**

Whole-lung perfusion correlated strongly between PCCT and V/Q-SPECT (*r* = 0.72, *p* < 0.05). Lobar correlations ranged from *r* = 0.62 to *r* = 0.85. PCCT yielded slightly higher perfusion values (mean PBV 0.50 ± 0.04) than V/Q-SPECT (0.49 ± 0.09). Bland–Altman analysis showed a bias of +0.015 (limits −0.13 to +0.16). Perfusion defect volumes correlated moderately (whole-lung *r* = 0.60, lobes *r* = 0.49–0.77, *p* < 0.05).

**Conclusion:**

PCCT-based perfusion imaging shows high concordance with V/Q-SPECT in this cohort, supporting its feasibility as a single-modality tool for functional and anatomical lung evaluation in CTEPH.

**Key Points:**

***Question*** *PCCT perfusion may address the unmet need for a combined functional and anatomical evaluation of CTEPH in a single examination*.

***Findings*** *PCCT showed strong correlation with V/Q-SPECT for lobar and whole-lung perfusion, with minimal bias and consistent quantitative agreement*.

***Clinical relevance*** *PCCT enables simultaneous high-resolution assessment of perfusion, vasculature, and parenchyma, potentially improving diagnostic confidence and streamlining CTEPH work-up in clinical practice*.

**Graphical Abstract:**

**Figure:**
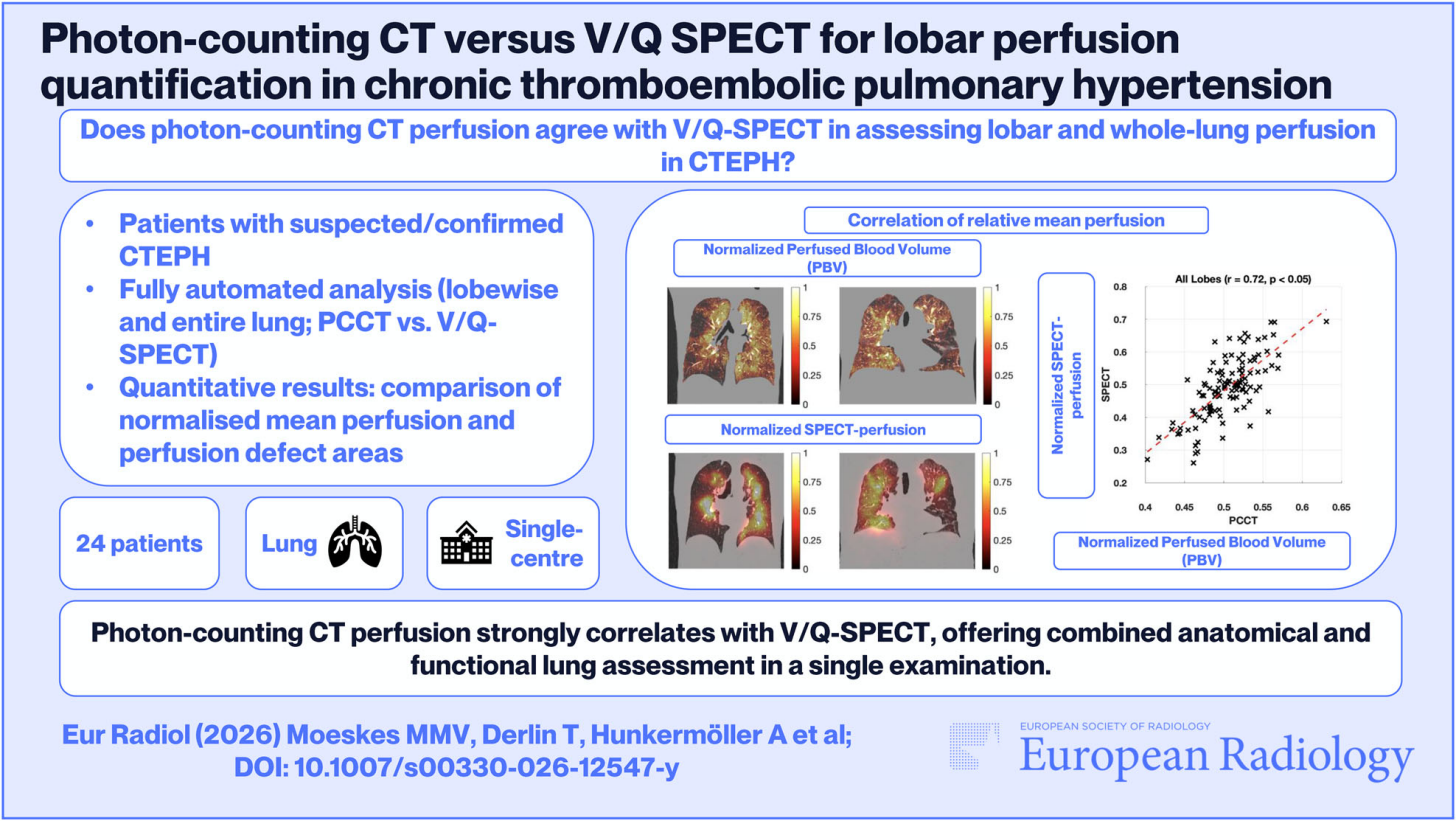

## Introduction

The diagnosis and management of chronic thromboembolic pulmonary hypertension (CTEPH) require a multimodal diagnostic strategy and interdisciplinary clinical collaboration [1, 2]. Epidemiological data indicate that a significant proportion of patients are diagnosed at advanced functional stages (New York Heart Association (NYHA) class III/IV), suggesting substantial underrecognition and delayed diagnosis of CTEPH [3–5].

Ventilation/perfusion single-photon emission computed tomography (V/Q-SPECT) remains the recommended first-line imaging modality for detecting chronic thromboembolic disease, characterised by persistent mismatched perfusion defects. Reported sensitivity and specificity of V/Q-SPECT range from 96% to 98% and 90% to 95%, respectively [6]. Despite its diagnostic utility, V/Q-SPECT has notable limitations, including underestimation of centrally located, non-occlusive thromboembolic lesions, limited spatial resolution, and insufficient anatomical detail of the pulmonary parenchyma [7, 8].

Dual-energy CT (DECT) has been extensively studied as an alternative functional imaging modality in CTEPH, offering iodine-based perfusion maps that demonstrate good concordance with V/Q-SPECT and MRI [9–15]. DECT allows combined CT pulmonary angiography and lung perfusion assessment in a single acquisition and can accurately localise perfusion abnormalities at the segmental and lobar level, which is crucial for operability assessment and treatment planning in CTEPH [16, 17]. Recent DECT studies have shown that perfused blood volume (PBV) correlates with disease severity, pulmonary vascular remodelling, and treatment response following balloon pulmonary angioplasty [12–14].

Photon-counting computed tomography (PCCT) represents an emerging imaging technology that utilises novel energy-resolving detector architecture to provide intrinsic spectral data, submillimetre spatial resolution, and enhanced radiation dose efficiency, yielding superior image quality compared to conventional energy-integrating detector systems and DECT [18–20]. These technical advances enable more precise iodine quantification and improved depiction of small vessels and peripheral perfusion abnormalities. Recent work has demonstrated that PCCT iodine maps can provide detailed perfusion assessment in CTEPH, revealing microvascular abnormalities and showing strong agreement with V/Q-SPECT [21–23]. In addition, a recently developed PCCT protocol enables the simultaneous evaluation of pulmonary perfusion, ventilation, vascular anatomy, and parenchymal morphology within a single examination [20]. Quantitative lobar perfusion assessment is clinically relevant in patients with CTEPH, as treatment decisions often require assigning perfusion defects to individual lobes for surgical endarterectomy planning, interventional procedures such as balloon pulmonary angioplasty, and longitudinal follow-up [16, 17]. A reliable lobar perfusion metric derived from CT may obviate the need for additional perfusion imaging in centres with PCCT availability, especially when combined with high-resolution angiography for characterisation of proximal disease distribution [24, 25]. This study forms part of a larger research project evaluating ventilation and perfusion imaging in CTEPH using PCCT. The present manuscript focuses specifically on quantitative perfusion assessment using PBV maps, while the ventilation analysis will be reported separately.

Accordingly, this study aimed to evaluate whole-lung and lobar agreement between PCCT-derived PBV maps and V/Q-SPECT perfusion in patients with suspected or confirmed CTEPH, and to investigate technical factors contributing to inter-modality differences.

## Materials and methods

### Study cohort

This retrospective, single-centre study included 24 patients undergoing evaluation for CTEPH with PCCT who also underwent V/Q-SPECT during the same study period (October 2021 to December 2024).

The study period coincided with the clinical rollout of PCCT at our institution, during which several vendor software and reconstruction updates were introduced. Although these updates were associated with incremental improvements in PBV image quality, PBV maps generated earlier in the study period provided sufficient image quality for quantitative analysis and demonstrated high concordance with V/Q-SPECT after normalisation.

**Fig. 1:**
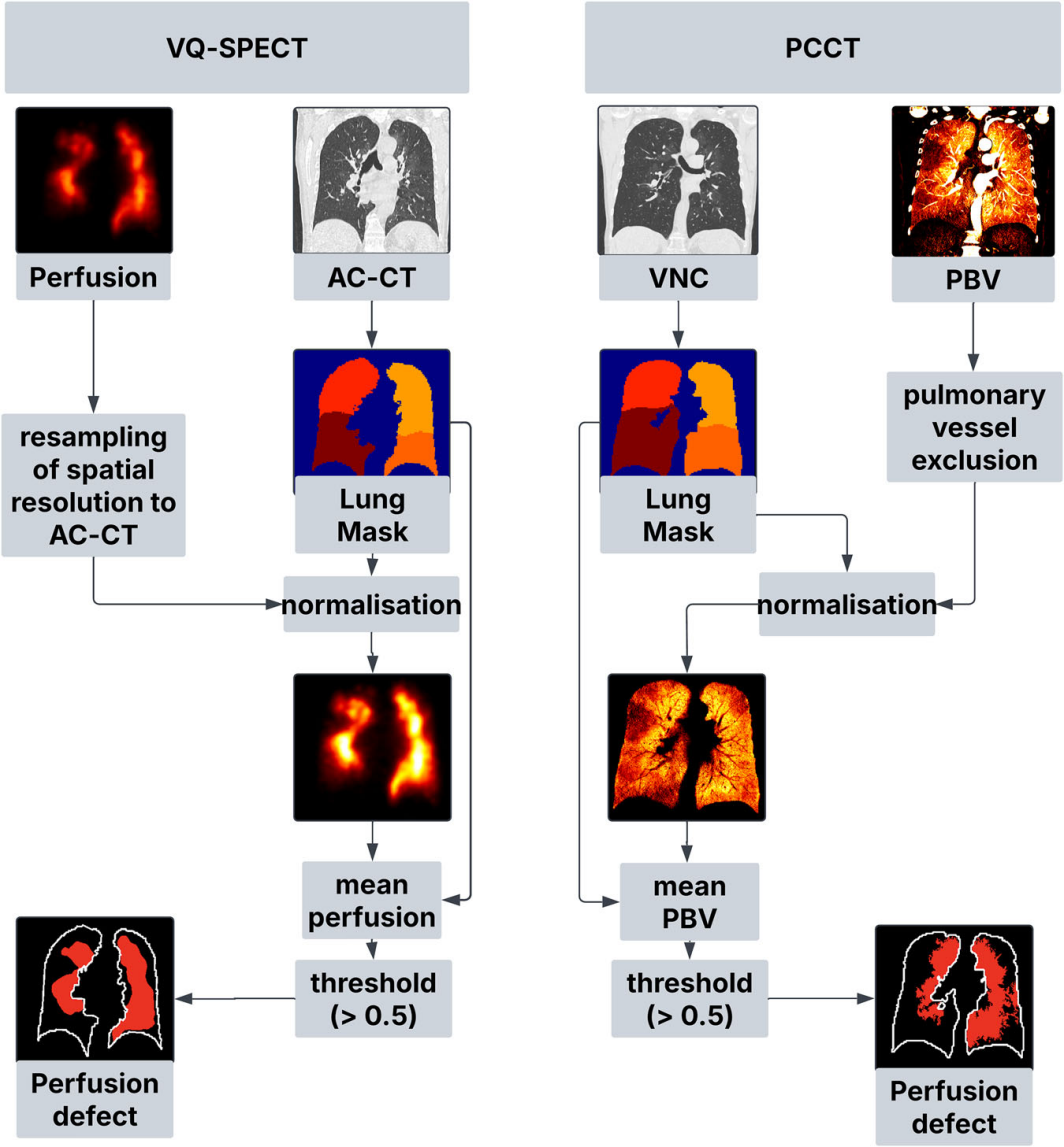
Image processing workflow for quantification of lobar mean perfusion and perfusion defect percentage from PCCT and V/Q-SPECT images. Lung and lobar segmentation were performed on inspiratory PCCT virtual non-contrast (VNC) and attenuation-correction CT (AC-CT) images using TotalSegmentator. Pulmonary vessels were excluded from PBV maps using a threshold-based vessel mask to enable comparison with V/Q-SPECT-derived perfusion. For inter-modal comparison, perfusion data from both imaging techniques were standardised using *z*-score normalisation (95% confidence interval). Mean PBV as a surrogate for lung perfusion was calculated for each lobe and the entire lung and used to derive lobar and whole-lung perfusion defect volumes

All individuals provided written informed consent, and the requirement for ethics approval was waived by the institutional review board (No. 10724_BO_K_2023). The initial cohort consisted of 10 females and 14 males. One female was excluded due to undergoing pulmonary thromboendarterectomy between the two imaging sessions (Fig. 1). Thus, 23 patients were included in the final analysis. The median age at the time of imaging was 69.8 years (absolute range: 45–84 years), with a mean age of 67.9 ± 10.7 years. PCCT and V/Q-SPECT were performed within a median interval of 3 days (range: 0–11 days). Patients were classified as having either suspected CTEPH under initial evaluation or confirmed CTEPH during further work-up or follow-up in a multidisciplinary setting. This small single-centre cohort therefore represents a real-world heterogeneous case mix including confirmed CTEPH, other forms of pulmonary hypertension and chronic thromboembolic disease without established pulmonary hypertension.

### Image acquisition

#### PCCT

A total of 23 CT scans acquired using a PCCT (Naeotom Alpha, Siemens Healthineers) were included. Two scan protocols optimised for lung parenchymal contrast were employed: a ventilation/perfusion CT (VQCT) protocol with paired inspiratory–expiratory acquisitions (*n* = 12) and a standard CT pulmonary angiography (CTPA) protocol with a single inspiratory acquisition (*n* = 11). The VQCT protocol followed a previously published approach [20]. All examinations were performed with patients in the supine position under standardised respiratory instructions. Iodinated contrast material (Iomeprol 400 mg I/mL; Iomeron 400, Bracco) was administered intravenously via an antecubital vein using a power injector. Bolus tracking was performed in the left ventricle in accordance with institutional standard practice to ensure robust opacification of the pulmonary arterial tree and to approximate peak parenchymal lung enhancement, largely independent of proximal vascular obstruction. Right-arm injections were preferred when feasible, but the injection side was not strictly standardised and was accounted for in the artefact analysis. PBV maps were reconstructed from the contrast-enhanced data using quantitative iterative reconstruction level 2 (QIR 2) with a QR40 kernel (section thickness 1.0 mm, increment 0.7 mm). Details of the scan parameters are provided in Table 1. The CTPA derived from the inspiratory PCCT scan enabled high-resolution visualisation of the pulmonary vasculature and detection of thromboembolic filling defects, while PBV maps were used for regional lung perfusion analysis [20]. The overall image processing workflow is illustrated in Fig. 1.

**Table 1:** Overview of the two CT scan protocols used in this study

	VQCT	CT pulmonary angiography (CTPA)
Patient position	Supine	Supine
Series information	inspiration/expiration	inspiration
Scan location	Chest	Chest
Scanning plane	Transverse	Transverse
Image quality level	67	40
QIR level	2	2
Tube voltage (kV)	120	120
Gantry rotation time (s)	0.25	0.25
Pitch	1.4	1.5
Tube current	CareDose4D	CareDose4D & care keV
Contrast material administration	50 mL contrast agent (Iomeron 400; Bracco) plus 50 mL NaCl at 3.5 mL/s; Phase 1: 80% contrast agent/20% NaCl; followed by saline flush	50 mL contrast agent (Iomeron 400; Bracco) plus 50 mL NaCl at 3.5 mL/s; Phase 1: 80% contrast agent/20% NaCl; followed by saline flush + 50 ml NaCl
Bolus tracking location	left ventricle	left ventricle
Bolus tracking threshold (HU)	100	100
Fixed trigger delay (inspiration) (s)	10	10
Fixed trigger delay (expiration) (s)	300	./.
Section thickness (mm)	1.0	1.0
Section interval (mm)	0.7	0.7

The technical parameters for both VQCT and CTPA protocols were comparable. The VQCT protocol included paired inspiratory and expiratory scans to capture ventilation and perfusion (only perfusion was evaluated in this study), whereas the CTPA protocol consisted of a single inspiratory-phase scan only

The use of both VQCT and CTPA protocols reflects clinical routine during the institutional implementation phase of PCCT and was accepted to maximise the sample size. Given the limited number of examinations in each subgroup, no formal statistical comparison between protocols was performed. Radiation dose metrics, including volume CT dose index (CTDIvol) and dose–length product (DLP), were recorded and converted to mSv for all examinations and reported as mean and standard deviation.

#### V/Q-SPECT

V/Q-SPECT was performed using a standardised protocol (GE Discovery NM/CT 670). Perfusion imaging was obtained following intravenous injection of technetium-99m-labelled human serum albumin microspheres, and ventilation imaging was conducted with Technegas inhalation [26]. Acquisitions were performed according to institutional standard-of-care protocols and were not modified for the purpose of this retrospective analysis. Tracer and X-ray exposure were quantified as mean and standard deviation in MBq and mSv.

### Image processing

V/Q-SPECT images (original voxel size: 4.4 mm isotropic) were resampled to match the dimensions of the attenuation correction CT (voxel size: 0.97 mm × 0.97 mm × 1 mm). Lung and lobe segmentation was performed on inspiratory PCCT virtual non-contrast images (VNC) and attenuation-correction CT (AC-CT) images using TotalSegmentator [27]. Due to differences in voxel intensity distributions between PCCT and V/Q-SPECT, *z*-score normalisation was applied to the perfusion data for comparison. Before normalising PBV maps, vessels were excluded using a vessel mask derived from a grey value threshold of 15% of the intensity in the main pulmonary artery, which was defined a priori based on previous work and pilot experiments [28].

For *z*-score normalisation, voxel intensities within the lung parenchyma were clipped to the 95% confidence interval and subsequently linearly rescaled to the range [0, 1]. This interval was selected to preserve the central distribution of physiologically relevant perfusion values while mitigating the influence of extreme voxels attributable to image noise, partial-volume effects, or artefacts. To quantify the perfusion, relative mean PBV and V/Q-SPECT perfusion values were calculated for each lung lobe and for the entire lung, as well as median and standard deviation. Perfusion defect volumes were evaluated by systematically varying the threshold from 0.2 to 0.8 in 0.1 increments and applying these to the normalised V/Q-SPECT perfusion maps and PBV maps from PCCT. The percentage of perfusion defects was calculated by dividing the number of perfusion defect voxels by the total number of voxels within the respective lung mask. All lung and lobar segmentations and vessel masks were visually inspected by a thoracic radiologist with more than 20 years of experience (H.S.). Predefined failure criteria included gross mismatch of lung boundaries, missing lobes, or clear misassignment of lobar borders. No examination met these failure criteria or required exclusion.

### Statistics

Statistical analysis was performed on data from 23 patients using MATLAB R2024b (TheMathWorks). Descriptive statistics were reported as mean ± standard deviation and ranges. Pearson correlation coefficients were calculated to assess the agreement between PBV and V/Q-SPECT mean values, as well as between their respective perfusion defect maps. A Bland–Altman analysis of the relative mean perfusion was performed to assess bias and limits of agreement between the two modalities. Quantitative perfusion values derived from PBV and V/Q-SPECT were evaluated on a lobar basis. All analyses were considered descriptive and exploratory, and no formal adjustment for multiple comparisons was performed.

## Results

### Study population and imaging interval analysis

The initial cohort included 24 patients (10 females, 14 males). One female patient was excluded due to undergoing pulmonary thromboendarterectomy between the two imaging sessions, resulting in a final sample size of 23 patients. Among these, 11 patients were diagnosed with CTEPH based on evaluation by a multidisciplinary board, primarily using characteristic findings from V/Q-SPECT, PCCT, and/or confirmation via pulmonary angiography. Of the remaining 12 patients, 11 had pulmonary hypertension (PH) of non-CTEPH aetiology, and one was diagnosed with pulmonary embolism (PE) without evidence of PH.

### Perfusion correlation and contrast-related artefacts

PBV consistently demonstrated slightly higher values compared to V/Q-SPECT across all lobes. The Pearson correlation coefficient for whole-lung mean PBV was *r* = 0.72 (95% CI: 0.62–0.80), indicating strong concordance between PCCT and V/Q-SPECT measurements. At the lobar-level, correlation coefficients were *r* = 0.62 (95% CI: 0.29–0.82) in the left upper lobe (LUL), *r* = 0.86 (95% CI: 0.69–0.94) in the left lower lobe (LLL), *r* = 0.82 (95% CI: 0.61–0.92) in the right upper lobe (RUL), *r* = 0.62 (95% CI: 0.28–0.82) in the middle lobe (ML), and *r* = 0.69 (95% CI: 0.39–0.86) in the right lower lobe (RLL). The relatively lower correlations in the LUL and ML are likely attributable to contrast influx artefacts during PCCT acquisition and residual spatial misregistration between PCCT and V/Q-SPECT (Table 2). Image artefacts were observed in 8 of 23 patients overall: 6 of 8 were contrast-related venous influx artefacts, 1 of 8 was related to osteosynthesis material and 1 of 8 to a port catheter. Among the 23 patients, contrast medium was administered via the left arm in 15 patients and the right arm in 8 patients. Artefacts consistent with venous contrast influx into the superior vena cava were identified in 5 of 15 patients (33%) with left-sided and 2 of 8 patients (25%) with right-sided injections. These artefacts were independent of the scanning protocol and are presumed to result from anatomical flow dynamics rather than technical variation.

**Table 2:** Lobe-wise perfusion metrics and corresponding correlations of mean PBV and V/Q-SPECT-perfusion

Lobe	Mean PBV ± SD	Mean perfusion V/Q-SPECT ± SD	Median PBV	Median perfusion V/Q-SPECT	Correlation *r* (95% CI)	*p*-value
LUL	0.5343 ± 0.0294	0.5223 ± 0.0764	0.5276	0.5052	0.62 (0.29–0.82)	0.0014
LLL	0.4881 ± 0.0347	0.4782 ± 0.1107	0.4887	0.4760	0.86 (0.69–0.94)	< 0.001
RUL	0.4826 ± 0.0242	0.4531 ± 0.0762	0.4826	0.4644	0.82 (0.61–0.92)	< 0.001
ML	0.4973 ± 0.0293	0.4953 ± 0.0796	0.4980	0.4928	0.62 (0.28–0.82)	0.0017
RLL	0.5224 ± 0.0293	0.5002 ± 0.1127	0.5238	0.5344	0.69 (0.39–0.86)	< 0.001
All lobes	0.5049 ± 0.0353	0.4898 ± 0.0939	0.5073	0.4943	0.72 (0.62–0.80)	< 0.001

Mean and median normalised PBV and V/Q_SPECT-perfusion values for each lung lobe were derived from PCCT and V/Q-SPECT after *z*-score normalisation to the 95% confidence interval and scaling to [0, 1]. PCCT consistently demonstrated slightly higher perfusion values compared to V/Q-SPECT across all lobes. Pearson correlation coefficients (*r*) and associated *p*-values revealed moderately strong to strong inter-modality agreement at the lobar-level, with all correlations reaching statistical significance (*p* < 0.05)

**Fig. 2:**
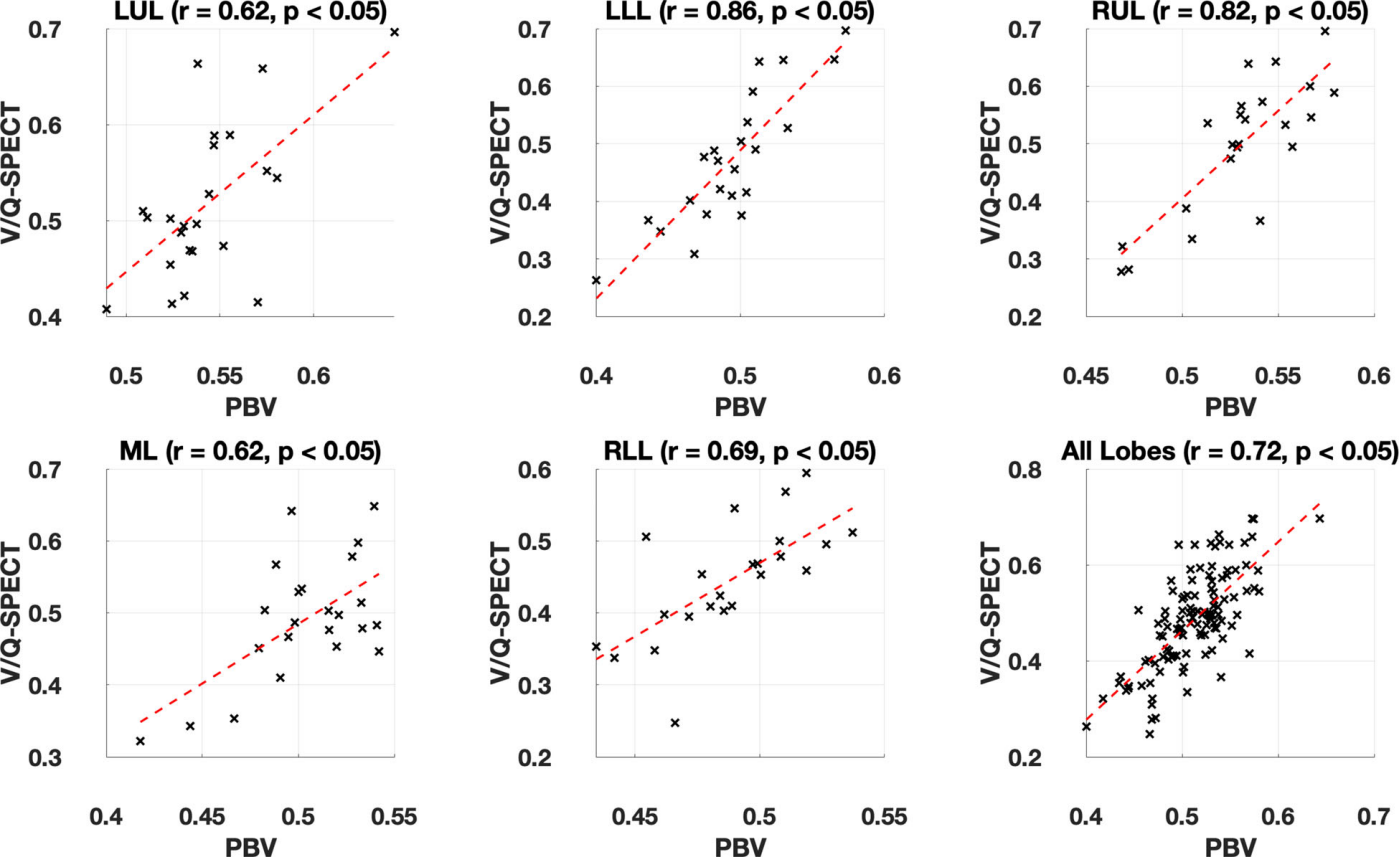
Lobar correlation of normalised mean PBV and V/Q-SPECT perfusion for all five lobes in 23 patients (115 lobar data points). There was a strong correlation (*r* = 0.72, 95% CI: 0.62–0.80) in whole-lung perfusion between PCCT and V/Q-SPECT, indicating good overall agreement between the two methods. At the lobar level, the LUL exhibited a moderately strong correlation (*r* = 0.62), notably lower than the LLL (*r* = 0.85). The reduced correlation in the LUL is likely due to contrast-related artefacts resulting from left-sided contrast administration during PCCT, which was observed in five of the 23 patients. In the right lung, the upper lobe (RUL) showed a strong correlation (*r* = 0.83), which exceeded those of the ML (*r* = 0.62) and the RLL (*r* = 0.69). The reduced correlations in ML and RLL may be due to spatial misalignment between PCCT and V/Q-SPECT. While V/Q-SPECT is acquired over several minutes during free breathing, the CT scan used for attenuation correction and lobar segmentation is acquired during a single mid-inspiratory breath hold. This temporal and respiratory phase mismatch can cause registration errors, particularly in regions like the ML and RLL, where adjacent lobe boundaries are more prone to displacement caused by breathing

Reduced correlation coefficients in the ML and RLL are likely due to spatial misregistration inherent to V/Q-SPECT imaging. Unlike PCCT, V/Q-SPECT acquisition is performed during free breathing over several minutes, which can result in spatial discrepancies when compared to the mid-inspiratory CT scan used for attenuation correction and anatomical segmentation. These discrepancies are particularly pronounced in lobes that are oriented transversely—such as between the ML and RLL—where precise segmentation is more challenging. Additionally, the greater respiratory motion in the lower lobes exacerbates misalignment, further reducing inter-modality correlation between ML and RLL (Fig. 2).

**Fig. 3:**
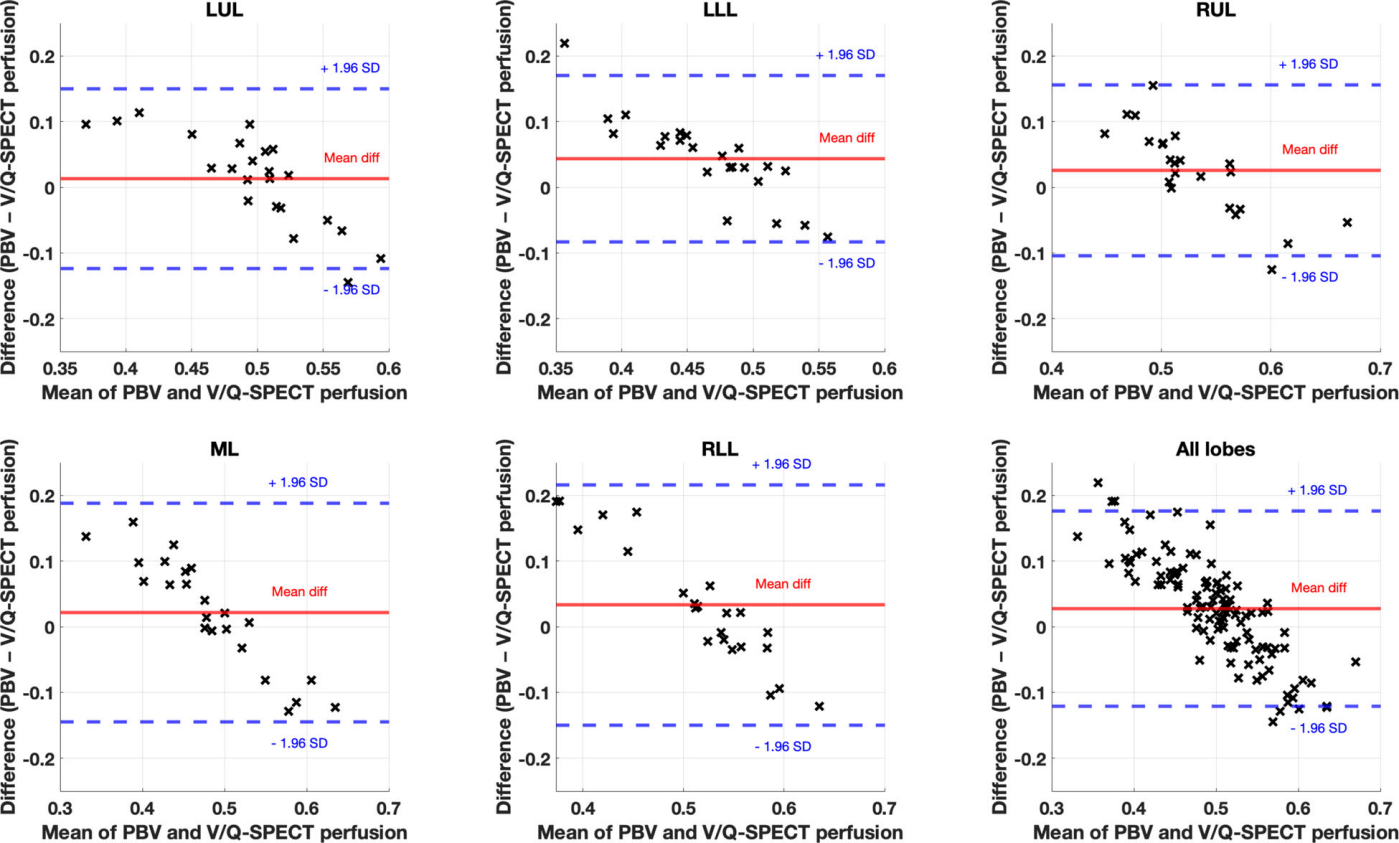
Lobe-wise analysis of perfusion agreement between PCCT and V/Q-SPECT. Bland–Altman plots show the difference in normalised lobar mean perfusion (PCCT minus V/Q-SPECT) against the corresponding lobar averages for all five lobes in 23 patients. Lobe-wise Bland–Altman analysis. The analysis revealed a mean bias of + 0.015, with limits of agreement ranging from –0.13 to + 0.16, suggesting a small but systematic overestimation of perfusion by PCCT. A negative trend with greater agreement at higher perfusion values can be observed, indicating a potential systematic proportional bias (see also Fig. 5)

Bland–Altman analysis revealed a mean bias of +0.015 (limits of agreement: –0.13 to +0.16), indicating a small but consistent overestimation of perfusion by PCCT. A negative trend in the Bland–Altman plot suggested a proportional bias, with better agreement at higher perfusion levels (Fig. 3).

**Fig. 4:**
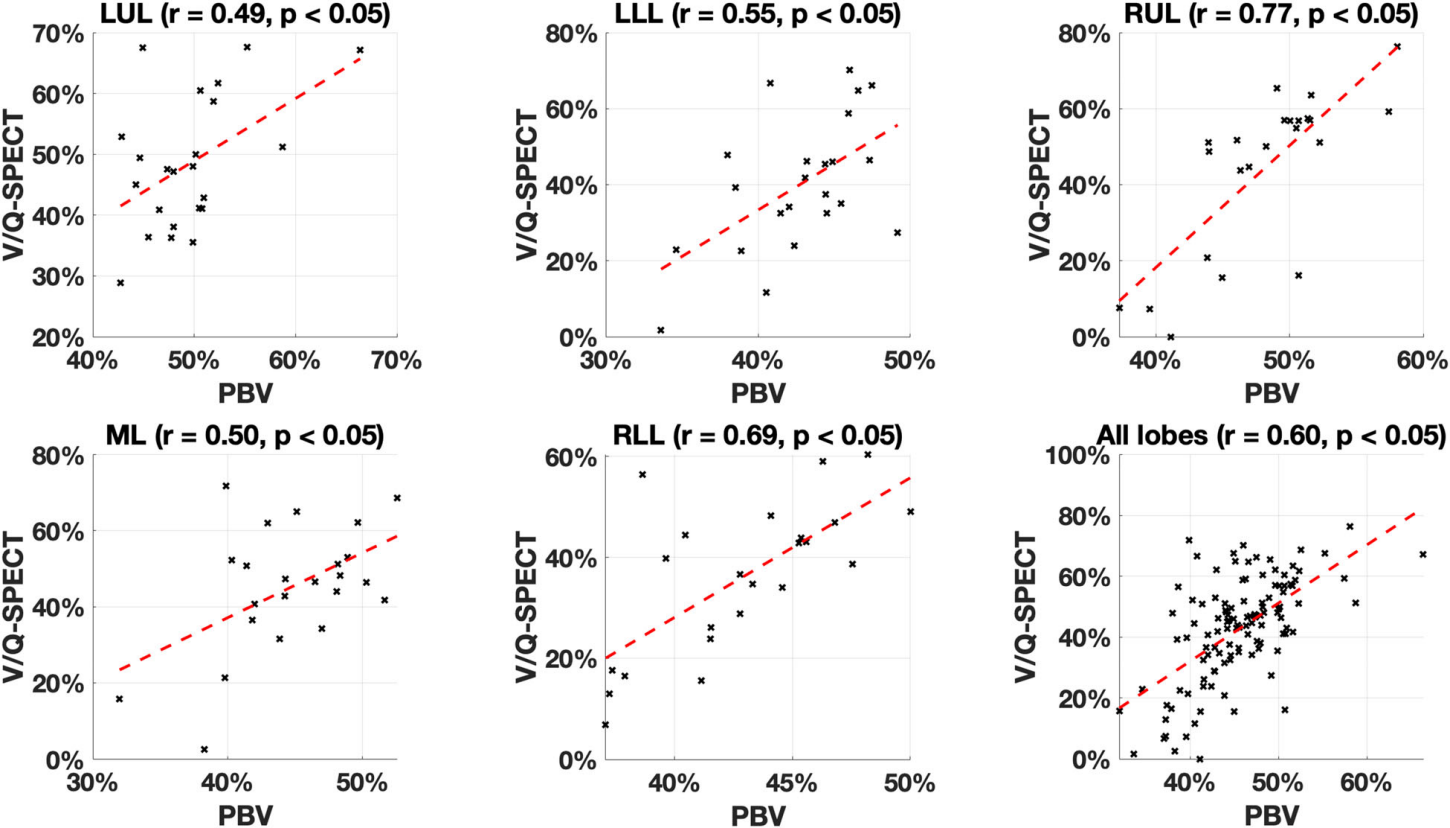
Correlation of perfused lung volume (%) per lobe and whole-lung (Threshold = 0.5). Lobar and whole-lung correlation of perfused lung volume (in percentage) derived from *z*-score–normalised perfusion maps in 23 patients. Correlations are generally slightly lower compared to the mean Perfusion analysis, ranging from 0.49 to 0.77. Yet, correlation for the entire lung was moderately strong (*r* = 0.60, *p* < 0.05). The lobar correlations of perfused lung volume demonstrated a pattern consistent with the mean perfusion correlation: the LUL showed lower correlation compared to the LLL. Similarly, correlations in the ML and RLL were lower than those observed in the RUL (see explanation in Fig. 2)

**Fig. 5:**
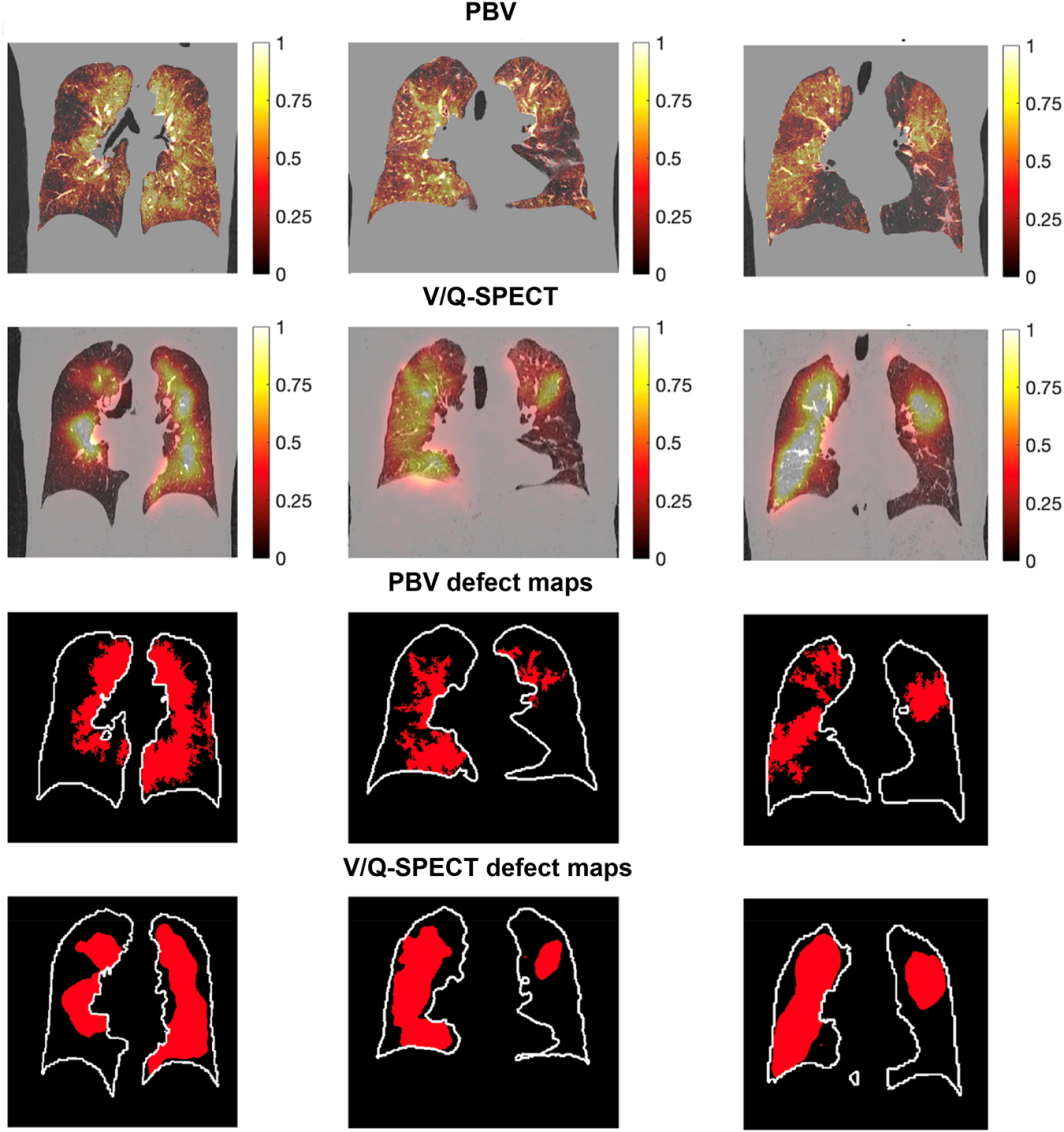
Representative examples of matched perfusion defects in CTEPH visualised by PCCT and V/Q-SPECT. Representative examples of three patients with CTEPH demonstrating matched perfusion defects in PCCT and V/Q-SPECT. The top rows display 5 mm coronal reformats of PBV maps from PCCT. The second row shows the corresponding V/Q-SPECT perfusion images, affinely registered to PCCT for visual comparison. The bottom rows present binary perfusion defect maps derived from *z*-score normalised perfusion images of both modalities, using a threshold of 0.5 (red areas indicate V/Q-SPECT-perfusion and PBV, respectively > 0.5). Patient 1 exhibits wedge-shaped perfusion defects in V/Q-SPECT, which correspond well with defects in both the normalised PCCT images and their defect maps. Patients 2 and 3 show predominant perfusion defects in the LLLs. Compared to V/Q-SPECT, PBV defect maps reveal a more heterogeneous or “scattered” appearance of preserved perfusion, reflecting its higher spatial resolution.

Perfusion defect volume exhibited a similar pattern of lobar correlation as mean PBV (Fig. 3). At the whole-lung level, PCCT and V/Q-SPECT demonstrated a moderately strong correlation in perfusion defect volume (*r* = 0.60, 95% CI: 0.25–0.81), with lobar correlations of *r* = 0.49 (95% CI: 0.10–0.75,) in the LUL, *r* = 0.55 (95% CI: 0.18–0.79) in the LLL, *r* = 0.77 (95% CI: 0.52–0.90) in the RUL, *r* = 0.50 (95% CI: 0.12–0.76,) in the ML and *r* = 0.69 (95% CI: 0.39–0.86) in the RLL (Fig. 4). Figure 5 shows representative images selected from the study cohort.

### Radiation dose

For PCCT examinations, the mean volume CT dose index (CTDIvol) was 15.2 ± 7.5 mGy (range, 5.3–36.8 mGy), and the mean dose–length product (DLP) was 262.5 ± 93.1 mGy·cm (range, 119–512 mGy·cm). Using an adult chest conversion coefficient of k = 0.014 mSv/(mGy·cm), this corresponds to an estimated effective dose of approximately 3.68 ± 1.30 mSv (range, 1.67–7.17 mSv). For the low-dose CT component of V/Q-SPECT, mean CTDIvol was 2.0 ± 1.0 mGy, mean DLP was 84.1 ± 40.9 mGy·cm, corresponding to an estimated effective dose of approximately 1.18 ± 0.57 mSv. Mean administered activities were 151.6 ± 6.7 MBq for ventilation imaging (Technegas) and 141.0 ± 20.4 MBq for perfusion imaging (Tc-99m MAA), corresponding to estimated effective doses of 2.27 ± 0.10 mSv and 1.55 ± 0.22 mSv, respectively.

## Discussion

This study demonstrates that PCCT-based perfusion imaging shows a high degree of concordance with the current reference standard, V/Q-SPECT, in patients with suspected or confirmed CTEPH. Strong correlations were observed for both whole-lung perfusion (*r* = 0.72) and lobar perfusion (range: *r* = 0.62–0.85), supporting the potential of PCCT-derived PBV maps as a promising quantitative tool for functional lung assessment in this patient population. Importantly, our study introduces a fully automatic, quantitative, lobar-based comparison of PCCT and V/Q-SPECT. By providing reproducible numerical metrics, PCCT enables objective validation of perfusion patterns, offering greater reliability than qualitative reader-dependent assessments. However, given the small, heterogeneous single-centre cohort and the exploratory nature of our analysis, these findings should be regarded as preliminary and hypothesis-generating rather than establishing diagnostic interchangeability between PCCT and V/Q-SPECT.

Our findings extend prior evidence from DECT studies, where iodine-based PBV maps showed strong diagnostic performance in CTEPH and other pulmonary vascular diseases [12–15]. In contrast to DECT, PCCT provides intrinsic spectral information without the need for dual-source hardware. The underlying detector physics of photon-counting systems allow for smaller detector elements, reduced electronic noise and more efficient use of the X-ray spectrum, which can be leveraged to generate high-resolution PBV maps at lower radiation doses than conventional DECT. Recent PCCT studies further highlight its advantages over DECT: Remy-Jardin et al demonstrated that PCCT iodine maps not only matched but, in some cases, exceeded the diagnostic accuracy of V/Q-SPECT in CTEPH, particularly for segmental and subsegmental defects [21]. Kerber et al [22, 23] confirmed that PCCT can provide comprehensive functional and anatomical information in a single examination, supporting its potential role as a one-stop modality in the diagnostic work-up of pulmonary hypertension. Our results add to this growing body of evidence by providing a lobar-level, fully quantitative comparison with V/Q-SPECT in a real-world CTEPH/PH cohort.

Clinically, these findings are significant as PCCT offers several distinct advantages. In contrast to V/Q-SPECT, PCCT enables high-resolution anatomical imaging, detailed visualisation of pulmonary vasculature and parenchyma, and simultaneous functional assessment of perfusion in a single scan. This integrated structural-functional capability may improve diagnostic confidence, particularly in complex cases where perfusion defects coincide with anatomical abnormalities. While PCCT offers distinct technical advantages, it is important to acknowledge that it cannot address the clinical issue of late CTEPH diagnosis on its own. Rather than solving underrecognition, PCCT contributes by consolidating anatomical and functional imaging. Recent studies specifically in CTEPH patients have demonstrated that PCCT iodine maps can uncover microvascular abnormalities [21] and show strong agreement with V/Q-SPECT for perfusion assessment [22, 23]. This is especially relevant for detecting proximal or segmental disease, which has direct implications for surgical eligibility and long-term outcomes [24, 25, 29].

Currently, PCCT-derived PBV maps should be considered a complementary imaging tool in centres with PCCT availability, potentially simplifying diagnosis and follow-up of CTEPH and other pulmonary hypertension entities.

Future applications may benefit from the integration of semi-automated quantitative tools, such as the Qanadli score [24] and right-to-left ventricular (RV/LV) ratio measurements [30, 31], to further enhance diagnostic precision and prognostication in CTEPH imaging. In addition, the development of standardised PCCT protocols for CTEPH—covering contrast administration, acquisition timing and reconstruction settings—may further reduce inter-patient variability and improve comparability across centres.

Comparable diagnostic performance has also been reported for dual-energy CT (DECT), where iodine-based perfusion imaging demonstrated good agreement with perfusion MRI and V/Q-SPECT in pulmonary vascular diseases [9, 10]. Supporting this, Tang et al showed in a canine model of CTEPH that DECT-derived perfusion defects correspond well with V/Q-SPECT findings, further supporting the validity of CT-based perfusion imaging in thromboembolic disease [11]. Compared with dual-energy CT, PCCT offers improved dose efficiency, intrinsic spectral data, and higher spatial resolution, with potential advantages for assessing subsegmental perfusion and microvascular abnormalities.

The moderate overestimation of perfusion by PCCT (mean bias +0.015) was observed, consistent with previous reports that iodine-based perfusion metrics may be affected by vascular signal contamination [20, 32]. Our application of vessel exclusion masks reduced this bias to some extent, though residual overestimation suggests either incomplete suppression or inherent differences in tracer kinetics compared to technetium-99m in V/Q-SPECT. Bland–Altman analysis further revealed a proportional bias, with improved agreement at higher perfusion levels. This indicates that PCCT may perform more consistently in well-perfused regions, likely due to both physiological variability and modality-specific sensitivity in low-flow detection.

Venous contrast influx artefacts emerged as an important practical limitation of PCCT-based PBV imaging in our cohort. We observed artefacts in 8 of 23 patients, 6 of which were contrast-related venous influx patterns and 2 related to hardware (osteosynthesis material and port catheters). Contrast-related artefacts occurred with both left- and right-arm injections, and in one case, an anomalous pulmonary venous drainage into the superior vena cava contributed to the pattern. These findings underline that venous inflow and variant anatomy can substantially distort PBV maps and thereby lower lobar correlations, particularly in the upper lobes. While our study mitigated these effects by vessel masking and *z*-score normalisation, more targeted protocol optimisation (e.g. preferential right-arm injection, adapted timing) and advanced correction algorithms will be required to further reduce the impact of venous influx artefacts in routine practice.

**Fig. 6:**
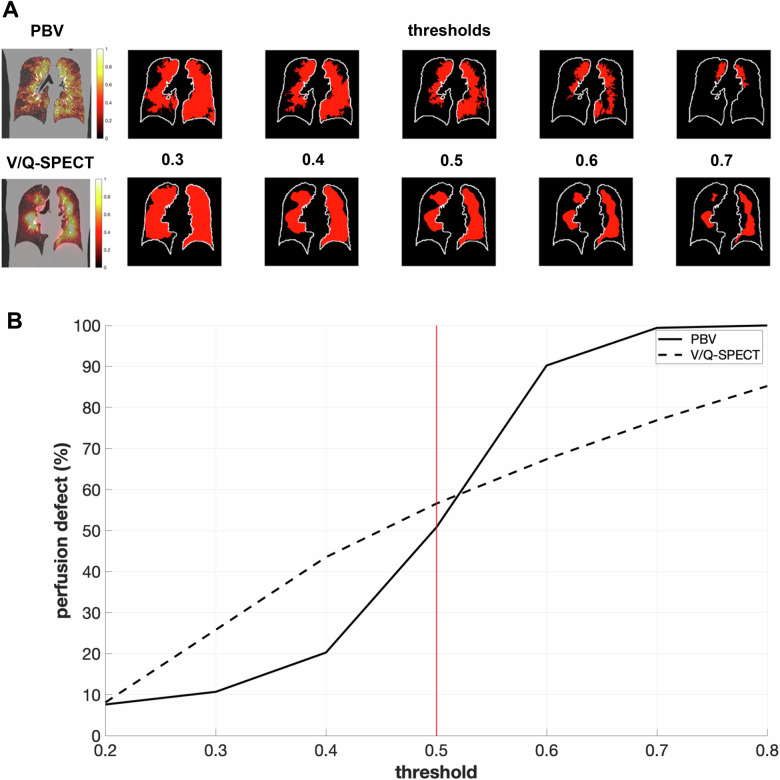
Influence of threshold on perfusion volume defects. **A** Effect of thresholding on perfusion defect maps derived from PCCT (top row) and V/Q-SPECT (bottom row) in a representative patient, using *z*-score–normalised perfusion maps scaled to the range [0, 1]. A threshold near 0.5 yields the highest visual concordance between the two modalities; both lower and higher thresholds introduce pronounced discrepancies. **B** The percentage of perfused volume is plotted against threshold values ranging from 0.2 to 0.8 for both PCCT and V/Q-SPECT across all 23 patients. The PCCT response exhibits a sigmoidal trend, while the V/Q-SPECT curve remains nearly linear. The curves intersect at a threshold of approximately 0.52. This indicates that PCCT underestimates hypoperfusion below this point and overestimates it above. The intersection suggests that a threshold of 0.52 provides the best alignment between PCCT and V/Q-SPECT perfusion assessments for the chosen normalisation scheme

Perfusion defect volume analysis revealed a moderately strong intermodality correlation (*r* = 0.60), with the greatest agreement around a perfusion threshold of 0.5. This empirically selected threshold—corresponding to the intersection of normalised perfusion curves (Fig. 6)—offers a reasonable trade-off between sensitivity and specificity. The sigmoidal curve observed in PCCT-derived data suggests sharper contrast between normally and abnormally perfused regions, consistent with PCCT’s higher spatial and contrast resolution. In contrast, the more linear profile of V/Q-SPECT reflects a broader dynamic range but lower spatial differentiation. Despite these intrinsic differences, the similar lobar distribution of perfusion defects between modalities underscores the clinical validity of PCCT. Nevertheless, the use of heuristic thresholds and the lack of segment-level diagnostic accuracy analysis or a formal reader study mean that conclusions about lesion-level detectability remain tentative and require confirmation in larger, prospective cohorts.

Artefact analysis revealed two primary sources of image degradation: contrast-related venous influx artefacts in PCCT and respiratory misregistration in V/Q-SPECT.

Contrast artefacts were more frequent with left-arm injections, likely due to the anatomy of venous drainage into the superior vena cava. This finding highlights the need for standardised contrast administration protocols to reduce asymmetry and inflow effects in PBV maps. In V/Q-SPECT, the lower correlation in the middle and RLLs was attributed to respiratory motion and spatial mismatches inherent in free-breathing acquisitions. These limitations are well-documented in the literature [8, 33] and may be partially addressed by respiratory gating or improved registration techniques [34]. Taken together, these modality-specific artefacts emphasise that technical optimisation on both sides—PCCT and V/Q-SPECT—is essential when quantifying inter-modality agreement.

In summary, our findings support PCCT as a promising tool for functional lung imaging in patients with CTEPH. PCCT closely approximates the diagnostic performance of V/Q-SPECT while offering the advantage of a comprehensive, single-examination assessment of both lung structure and function. In addition, the fully automated analysis enables objective and reproducible quantification of perfusion and perfusion defect volumes.

### Limitations

Several limitations warrant consideration. First, this retrospective single-centre study included a relatively small and clinically heterogeneous cohort. Data acquisition occurred during the institutional rollout of PCCT (2021–2024), including multiple vendor software and reconstruction updates, which may limit generalisability. Protocol heterogeneity (VQCT and CTPA) and mixed acute and chronic clinical settings may have further increased measurement variability. The interval between PCCT and V/Q-SPECT was also variable; accordingly, residual temporal confounding cannot be completely ruled out. Second, although *z*-score normalisation enabled meaningful cross-modality comparisons, it is a simplification that does not account for all differences in image acquisition, tracer kinetics, and physiological distribution, and it relied on heuristic choices for vessel masking (15% of main pulmonary artery intensity), restriction to the central 95% of parenchymal voxels and empirically selected perfusion-defect thresholds, which were not formally optimised or externally validated. Third, functional ventilation data from PCCT were not analysed in this study, but their future integration could further enhance single-scan comprehensive evaluation. Fourth, we did not perform a structured qualitative image assessment with inter-reader agreement, nor a segment-level diagnostic accuracy analysis or multi-reader study, which would have added additional validity to the quantitative analysis.

### Conclusion

PCCT-derived perfusion mapping demonstrates high correlation with V/Q-SPECT across both whole-lung and lobar assessments in patients with suspected or confirmed CTEPH. Despite minor differences in absolute perfusion values and the presence of modality-specific artefacts, PCCT presents a viable, efficient, and more comprehensive imaging alternative. Further prospective multicentre studies using a standardised PCCT protocol are warranted to validate these findings in larger cohorts and to explore the integration of PCCT perfusion with other functional parameters such as ventilation and right heart hemodynamics.

## Abbreviations

AC-CT: Attenuation-correction CT
CTEPH: Chronic thromboembolic pulmonary hypertension
LLL: Left lower lobe
LUL: Left upper lobe
ML: Middle lobe
PBV: Perfused blood volume
PCCT: Photon-counting CT
RLL: Right lower lobe
VQCT: Ventilation/perfusion-CT
V/Q- SPECT: Ventilation/perfusion single-photon emission computed tomography
